# Dendritic fibromyxolipoma in the right inguinal and perineum regions: a case report and review of the literature

**DOI:** 10.1186/1746-1596-8-157

**Published:** 2013-09-20

**Authors:** Xing Jian Zhang, Song Zhou, Kai Nie, Da Feng Chen, Guo Ju Kui, Xue Hui Zhang

**Affiliations:** 1 Department of General Surgery, the 175th Hospital of PLA, Southeast Hospital Affiliated to Xiamen University, NO. 269, Zhanghua Middle Road, Zhangzhou 363000 Fujian Province, China; 2 Department of Pathology, the 175th Hospital of PLA, Southeast Hospital Affiliated to Xiamen University, NO. 269, Zhanghua Middle Road, Zhangzhou 363000 Fujian Province, China

**Keywords:** Dentritic fibromyxolipoma, The inguinal region, The perineum region

## Abstract

**Abstract:**

A 32-year-old woman presented with a slow-growing, painless, subcutaneous lesion in the right inguinal and perineum regions. The mass was 24.0 cm × 10.5 cm × 5.0 cm in size, well circumscribed, mobile, and rubbery. Microscopically, the resected mass was mainly composed by a proliferation of small spindle or stellate cells, variably admixed with mature adipose tissue, embedded within an abundant myxoid and collagenized stroma. Immunohistochemically, the spindle and stellate cells were strongly positive for vimentin, CD34, and bcl-2 antibodies but not for smooth muscle actin and desmin. The tumor was diagnosed as dendritic fibromyxolipoma based on the typical findings of histology and immunohistochemistry. Clinical follow-up of 9 months after surgery revealed no evidence of recurrence. We report the first case of dendritic fibromyxolipoma in the right inguinal and perineum regions and discuss the different diagnosis.

**Virtual slides:**

The virtual slide(s) for this article can be found here: http://www.diagnosticpathology.diagnomx.eu/vs/1313680868103019.

## Introduction

Dendritic fibromyxolipoma (DFML) is a rare benign soft tissue lesion that most commonly arises in the subcutis or muscular fascia of the head and neck, shoulders, calf, foot, or back in adult male patients. The characteristic histologic picture is described as an admixture of mature adipose tissue, spindle and stellate cells, and abundant myxoid stroma with prominent collagenization. These neoplasms typically show positive immunoreactivity for CD-34, bcl-2 and Vimentim. We describe a case of DFML in the right inguinal and perineum regions. To our best knowledge, female DFML in inguinal including the perineum region has not been reported.

### Clinical summary

**Figure 1:**
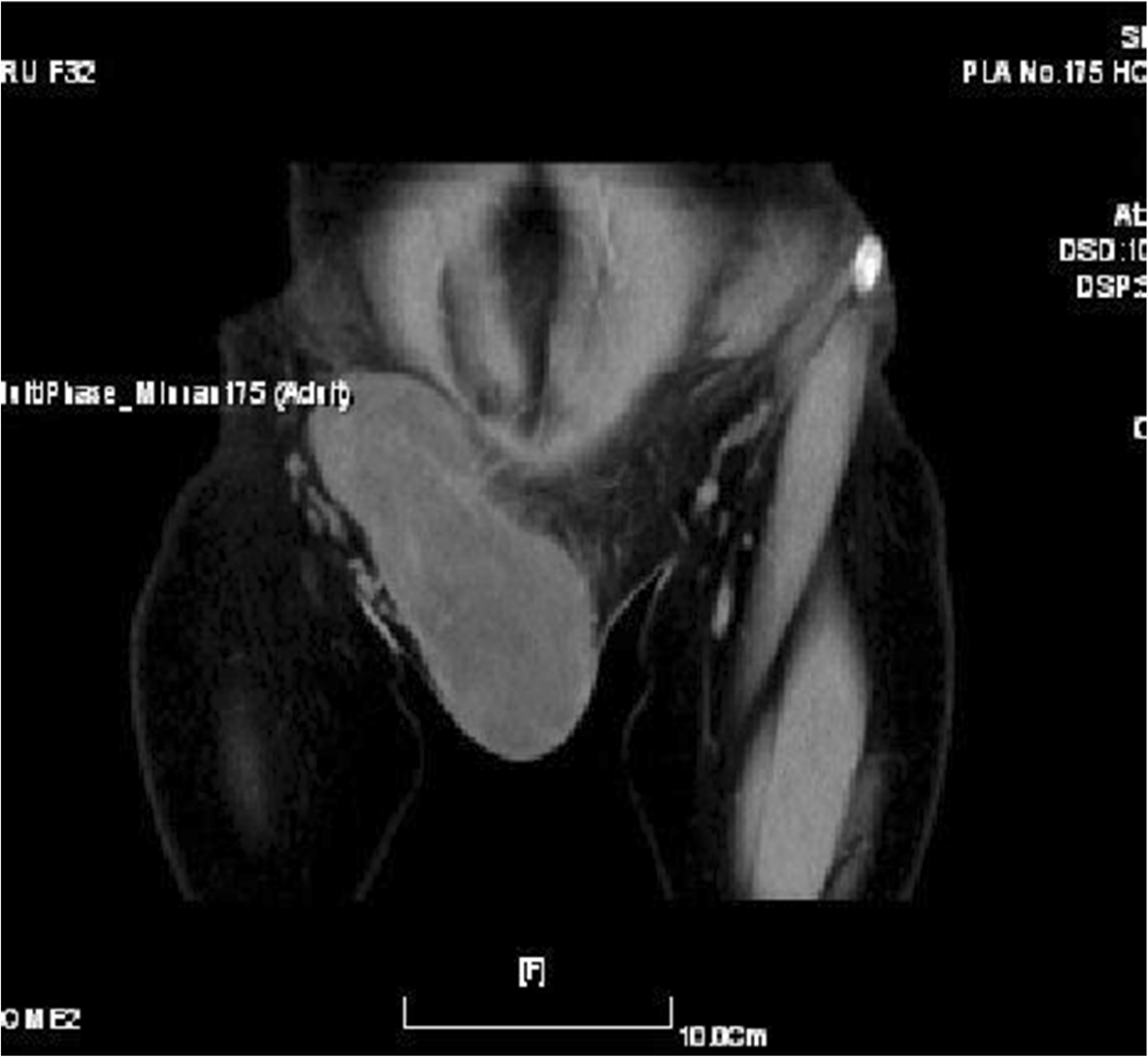
Computed tomography scan showed the tumor was located in the subcutis, well-demarcated, and isolated from the abdominal cavity.

**Figure 2:**
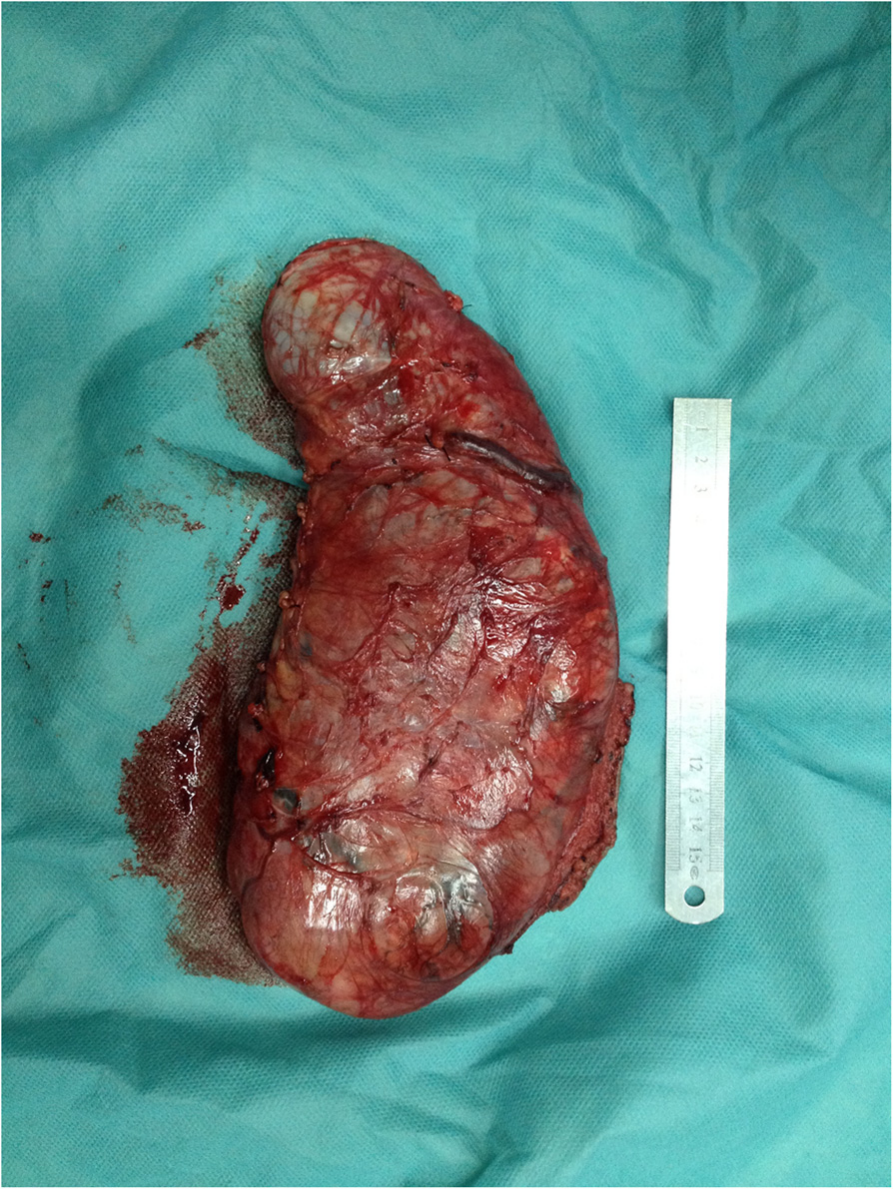
**Gross appearance of the tumor.** The tumor was soft and well-circumscribed by a thin fibrous capsule.

**Figure 3:**
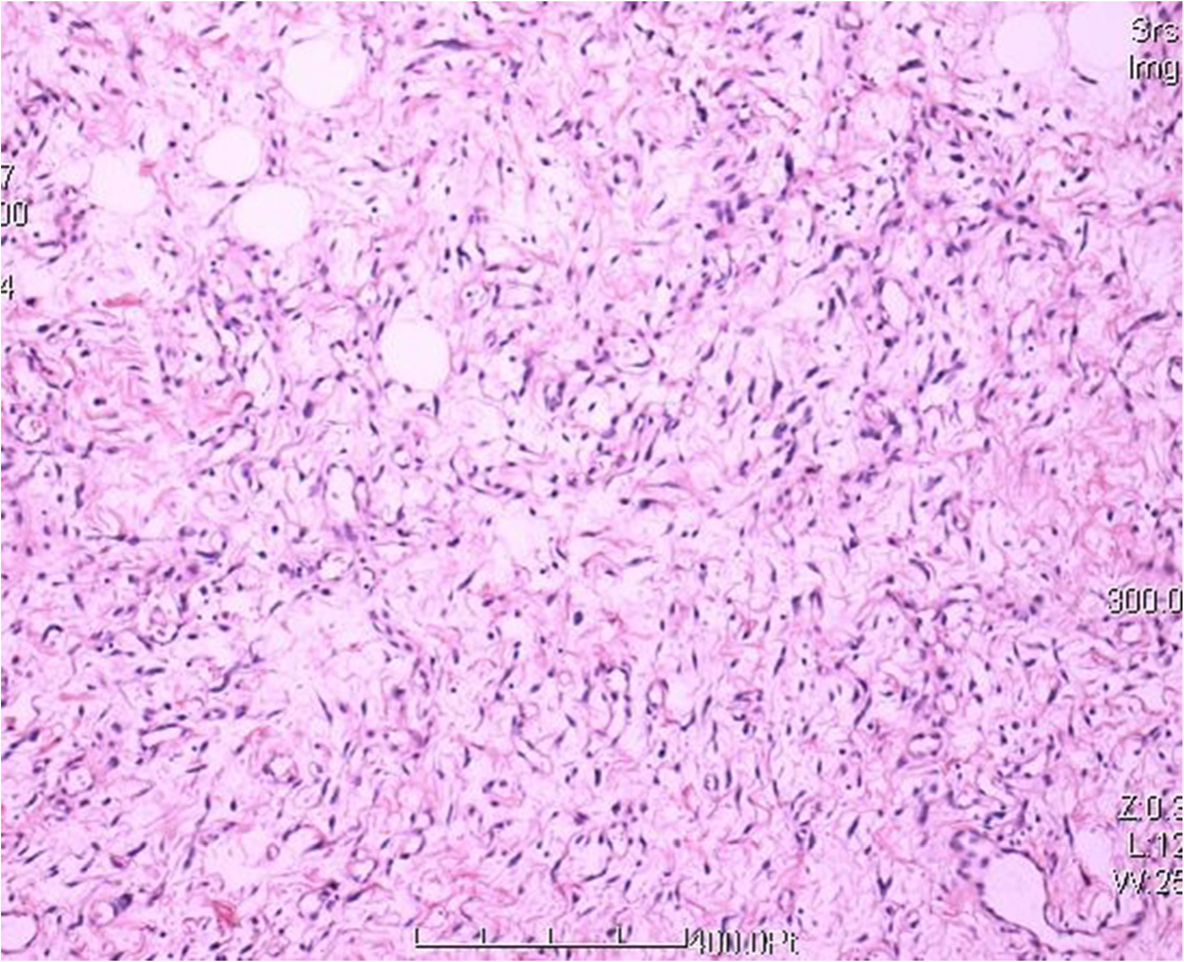
Histologic features of the lesion showed the tumor was composed by small spindle cells, variably admixed with mature adipose tissue, embedded within an abundant myxoid (HE 100×).

**Figure 4:**
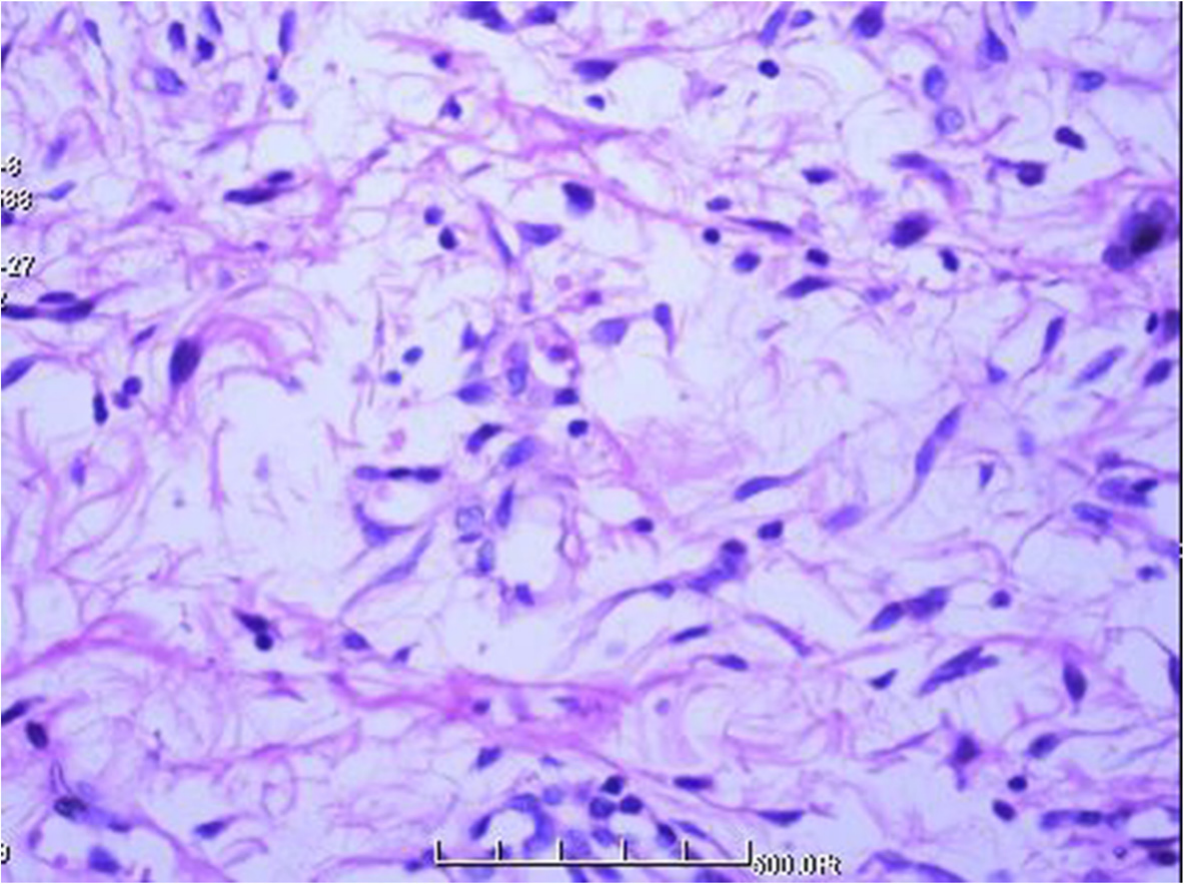
Higher magnification showing spindle and stellate cells with thin, dendritic cytoplasmic prolongations (HE 400×).

**Figure 5:**
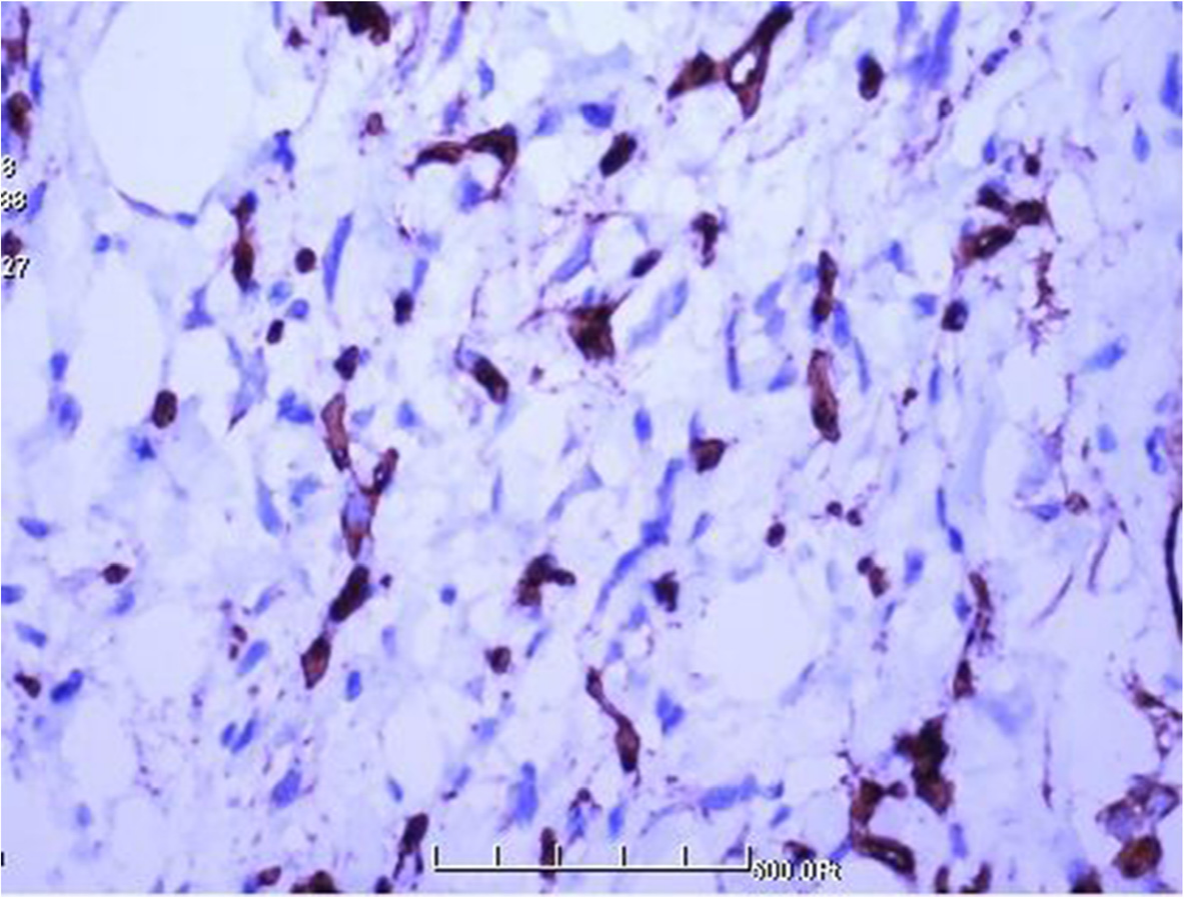
Strong immunoreactivity for CD34 (100×).

**Figure 6:**
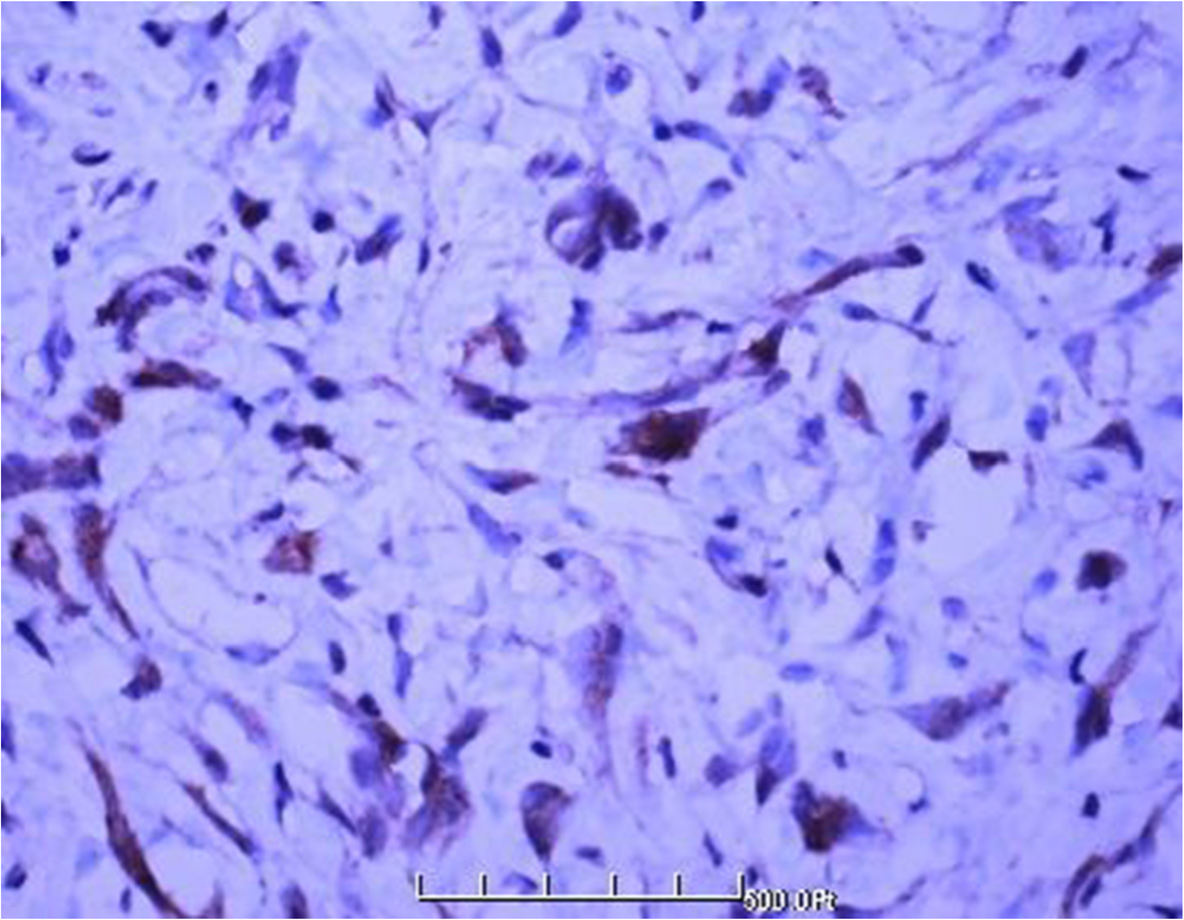
Strong immunoreactivity for bcl-2 (100×).

**Figure 7:**
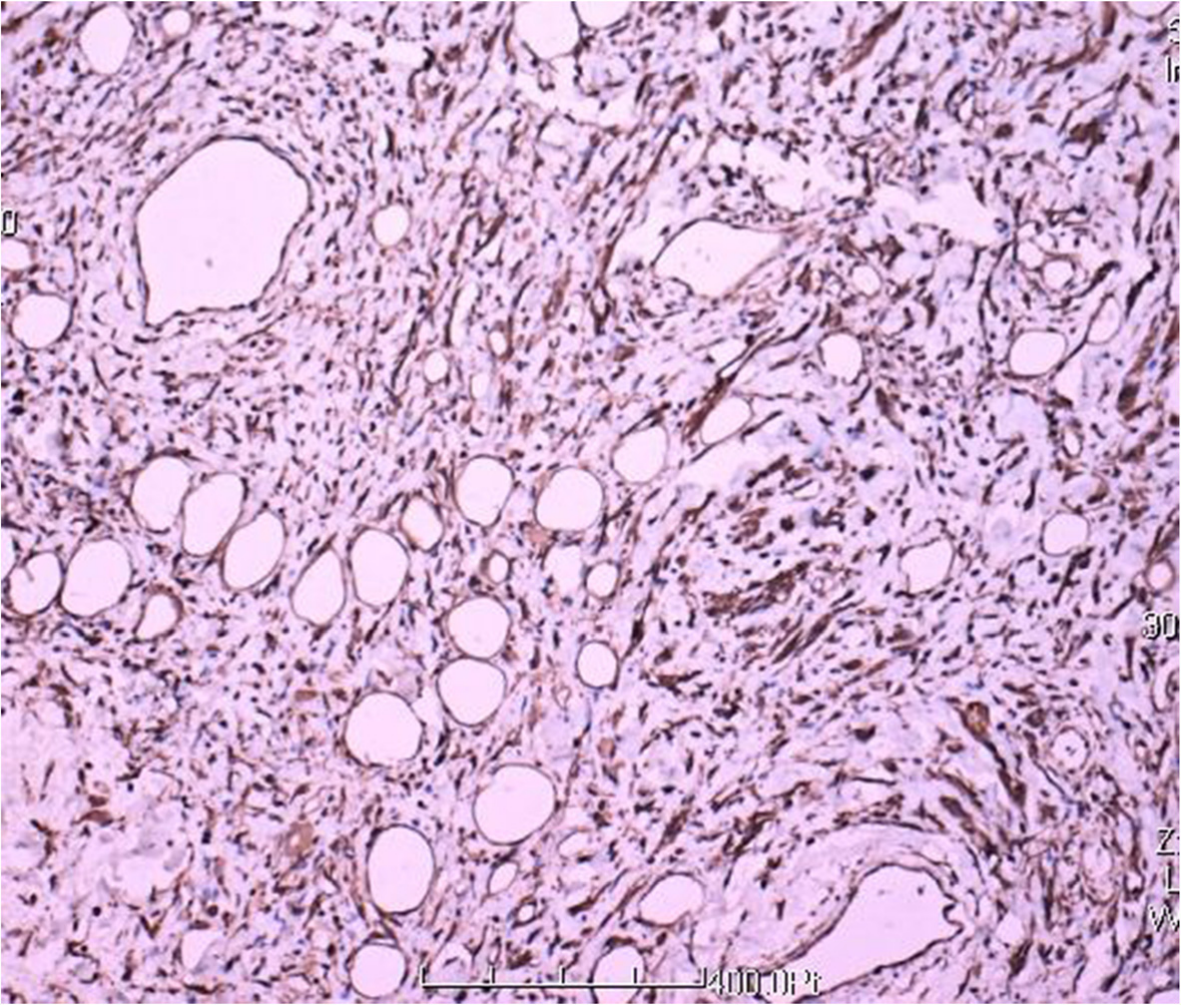
Strong immunoreactivity for vimentin (100×).

A 32-year-old woman was first seen by us 3 years after becoming aware of a painless subcutaneous mass in her right inguinal and perineum regions. Initially, the mass was in the right inguinal region, grew up slowly towards the perineum region. The mass was unreducible and could not become more prominent when coughed, strained, or stood up. The mass was grew fast with no obvious incentive in the past three weeks. On physical examination, the mass was subcutaneous, 20 cm × 10 cm × 7 cm in size, mobile, rubbery, unreducible. The transillumination test and auscultation for bowel sounds on the mass were negative. Her past and family histories were non-contributory. Laboratory data as well as tumor markers, such as CEA, CA19-9, were normal. Ultrasonography (US) revealed a well circumscribed, inhomogeneous mass with prominent vascularity, measuring about 19.7 cm × 9.2 cm × 6.8 cm, in the right inguinal and perineum regions. The mass originated from the right labium majus pudendi and extended to the low abdominal wall. No evidence was shown for a connection between the abdominal cavity and the mass. Also, computed tomography (CT) demonstrated a mixed density, well circumscribed mass in the same region. CT value of the mass ranged from −9 to 25 Hu, which revealed a soft tissue. Enhanced CT scanning revealed a heterogeneous, medium enhanced with prominent collagenization in the mass (Figure 1). Firstly, it was clinically considered to be an irreducible hernia. Then, a myxoid liposarcoma was considered to be the best preoperative diagnosis after combined with physical examination and radiographic outcomes. Surgical treatment was performed in August 2012. Rapid intraoperative pathological diagnosis revealed a benign mesenchymal tissue neoplasm and the complete local excision was carried out. Macroscopically, the excised tumor was 24.0 × 10.5 × 5.0 cm in size, soft and well-circumscribed by a thin fibrous capsule. The cut surface was yellow-gray and mucoid (Figure 2). Histologically, the tumors was mainly composed by a proliferation of small spindle or stellate cells variably admixed with mature adipose tissue embedded within an abundant myxoid and collagenized stroma. The spindle cells had a small hyperchromatic nuclei in which pleomorphism, atypia, or mitotic activity were extremely rare (Figures 3 and 4). Immunohistochemical staining revealed that the spindle and stellate cells stained strongly positive for vimentin, CD34, and bcl-2 antibodies, Stains for smooth muscle actin and desmin were negative (Figures 5, 6 and 7). The patient’s postoperative course was unremarkable. No evidence of the local recurrence or metastasis has been seen in the 9 months since excision.

## Discussion

Dendritic fibromyxolipoma is an uncommon benign soft tissue tumor that first reported by Suster et al. in 1998 in twelve patients [1]. Since then, only four cases of DFML, including ours, have been reported in the English literature and cited in Pub Med (Table 1). The mass more commonly arises in the subcutaneous tissue of subcutis or muscular fascia of the head and neck, shoulders, chest wall or back, and predominantly affects male adults. Although uncommon, there are still cases of DFML reported in the intramuscular and in the median nerve. Clinical follow-up in all available cases showed no evidence of recurrence or metastasis after surgical treatment [2-4].

**Table 1:** Clinical findings of DFML in the 15 patients reported in the Einglish literature

**Reference**	**Year**	**Sex/Age**	**Location size (cm)**	**Clinical follow-up**	**Immunohistochemical**
Suster [1]	1998	M/33	11-cm mass in left posterior shoulder acromium region	INA	CD34+, BCL-2+
		M/58	7.5 × 5.5 × 3.0 cm, right shoulder	NED, 7 year	CD34+, BCL-2+
		M/54	5.0 × 5.0 × 4.0 cm, right posterior neck	INA	CD34+, BCL-2+
		M/63	6.0 × 5.5 × 2.0 cm, upper back	INA	CD34+, BCL-2+
		M/66	8.0 × 3.5 × 2.5 cm, back of the neck	INA	CD34+, BCL-2+
		M/66	9.0 × 7.0 × 6.5 cm, back, posterior axillary fold	INA	CD34+, BCL-2+
		M/70	2.0 × 2.0 × 2.0 cm, face right nasal area	NED, 11 year	CD34+, BCL-2+
		M/73	7.0 × 5.5 × 2.5 cm, right posterior neck	NED, 13 year	CD34+, BCL-2+
		M/77	3.0 × 2.0 × 1.5 cm, back of neck	NED, 5 year	CD34+, BCL-2+
		M/79	3.5 × 3.0 × 2.5 cm, right chest wall	Died of meta static Carcinoma of giant cell carcinoma of the Lung 2 year after surgery	CD34+, BCL-2+
		M/81	3.5 × 3.0 × 3.0 cm, left chest wall, infraclavicula	NED, 5 year	CD34+, BCL-2+
		F/50	6.0 × 5.5 × 5.0 cm, right upper back	INA	CD34+, BCL-2+
Karim [2]	2003	M/73	13.0 × 8.0 × 5.5 cm, between the infraspinatus and deltoid muscles	NED, 8 mouths	CD34+, BCL-2 +
Dahlin [3]	2012	F/65	The median nerve in the left forearm	INA	CD34+, BCL-2 +
Al-Maskery [4]	2011	F/36	2.0 × 2.0 × 2.0 cm	NED, 2 years	CD34+, CD-99+
			in the lower lip		BCL-2 +

M, male; F, female; INA, information not available; NED, no evidence of recurrence.

The most striking histologic feature of DFML is an admixture of mature adipose tissue, spindle and stellate cells, and abundant myxoid stroma with prominent collagenization. Immunohistochemically, the vimentin and CD34 immunohistochemical stains accentuated the cell’s dendritic nature by revealing slender, complex cytoplasmic prolongations which are the main reason of it’s named [1]. The curative treatment for DFML is completely local excision. Recurrence or metastasis has never been reported in DFML patients after surgical treatment.

DFML should be differentiated from some benign lesions: spindle cell lipoma (SCL), solitary fibrous tumor(SFT), lipoblastoma, lipoblastomatosis, and nodular fascitis. Of the other tumor-like lesions, SCL is most likely to be confused with DFML. SCL is composed of a mixture of mature adipocytes and uniform spindle cells within a matrix of mucinous material traversed by a varying number of birefringent collagen fibers. It shares many features with DFML including age, male predilection, location, gross features. The signally similar clinical and histological feature of the lesions makes it difficult for distinguishing DFML from SCL [5]. Suster et al. emphasized the dendritic nature of the spindle cells, the plexiform vascular pattern, and the abundance of keloidal collagen as the three essential features in DFML, which were not commonly presented in SCL [1]. But recently studies revealed that some features, such as prominent vascular patterns, and short bipolar cytoplasmic extensions, also had been seen in SCL [2].

Other benign spindle cell tumor that should be distinguished from DFML is solitary fibrous tumor (SFT). SFTs which have a predilection for the thoracic cavity are rare fibrous neoplasms. Histologically, the tumor is characterized by a “patternless pattern” of short spindle cells with scant cytoplasm and bland cytologic appearance separated by strands of rope-like collagen, and a “hemangiopericytoma-like” pattern where the lesional cells are densest around small and medium ectatic and branching vessels [6]. The “hemangiopericytoma-like” vascular pattern and the lack of an adipose tissue component are two histologically features for distinguishing SFT from DFML [2]. Lipoblastoma and lipoblastomatosis are another two rare benign soft tissue mesenchymal tumours that may be confused with DFML. The tumours mainly occur almost exclusively in infants and children under the age of 3 years. The common microscopic features of lipoblastoma and lipoblastomatosis have been described as a mixture composed of immature lipoblasts,mature lipocytes,embedded in an abundant myxoid stroma. DFML could be easily distinguished from lipoblastoma and lipoblastomatosis by the patients age and the absence of lipoblasts [1,7]. Nodular fascitis is another lesion that should be differentiated from DFML. Nodular fascitis shows proliferating spindle cells embedded in a loosely textured myxoid and inflammatory stroma. Unlike DFML, the lesion is relatively well circumscribed but poor encapsulated. Immunohistochemically, the spindle cells are positive for muscle markers except desmin and are S-100 protein and CD-34 negative [8].

The distinction between DFML and malignancy, both of which can have a plentiful myxoid matrix, proliferation of capillaries and relatively large size, is more subtle and difficult. Among the malignant tumors, the possibility of a myxoid liposarcoma (MLS) should be seriously considered. MLS, a low grade malignancies, is the most common subtype of liposarcoma. MLS shares many features with DFML including a delicate plexiform vascular pattern, large size, and a myxoid matrix. But MLS is distinguished from DFML by the lower extremities fascial planes predilection, the infiltration of surrounding structures, and the presence of lipoblasts on higher magnification [1,9]. Furthermore, molecular studies had shown that MLS was characterized by the recurrent translocations t(12;16)(q13;p11) and, less commonly, t(12;22)(q13;q12), which fuse FUS or EWSR1, respectively, to DDIT3 on chromosome 12 gene [10]. Narendra et al. confirmed that FISH with DDIT 3 break-apart probe was a valuable adjunct in diagnosis or differential diagnosis of MLS [11].

DFML has never been described in the perineum region. DFML in the perineum region of females should be differentiated from perianal sepsis, lesions of anogenital mammary-like glands (AGMLG) and some relatively site-specific stromal tumours. Perianal abscess/sepsis is the most common cause of a mass in the perineum region and can be easily differentiated from DFML by its clinical presentation [12]. AGMLG are a newly recognized variant of cutaneous adnexal glands that are found in the anogenital area of both sexes, with characteristics of modified eccrine and apocrine glands. Lesions of AGMLG such as hidroadenoma papilliferum, apocrine cystadenoma, adenosis tumor, and extramammary Paget's diasease, show a typically striking homology with lesions in the breast. Histologically, the appearance of glandular structures and the histological homology to its counterpart breast lesions, helps distinguish lesions of AGMLG from DFML [13]. Some stromal tumours, including angiomyofibroblastoma, aggressive angiomyxoma, cellular angiofibroma, leiomyosarcoma, rhabdomyosarcoma and epithelioid sarcoma, can also be found in the perineum region [14,15]. Of these, angiomyofibroblastoma, aggressive angiomyxoma, leiomyosarcoma, rhabdomyosarcoma and epithelioid sarcoma can be easily distinguished from DFML by their histopathological and immunohistochemical findings. The distinction between DFML and cellular angiofibroma, both of which can have adipocytes in addition to the characteristic vascular network and connective tissue stroma, is more subtle and difficult. However, cellular angiofibroma lacks the dendritic processes and prominent myxoid component of DFML. In addition, the striking hyalinization of the blood vessel walls in cellular angiofibroma is another distinctive feature not found in DFML [2,16].

Even though DFML has been found for more than 14 years, the nosological position of DFML is still indeterminate. Suster et al. [1] initially stressed that DFML represented a transitional form between SCL and SFT. But recently studies by Fritchie et al. [17] on the molecular relationship with SCL and SFT had found that only SCL showed monoallelic or biallelic loss of 13q14(RB1), revealed that SCL had not relationship with SFT. Karim et al. [2] thought that DFML probably represents an peculiarly variant of myxoid SCL, based on their similarly clinical and pathological features.

## Conclusion

Dendritic fibromyxolipoma is very rare benign tumor. We report the first example of DFML in the right inguinal and perineum region. A diagnosis of DFML should be made by their microscopical and immunohistochemical features. DFML should be considered in the differenial diagnosis of lesions with spindle cell lipoma, solitary fibrous tumor, lipoblastoma, lipoblastomatosis, nodular fasciitis, and myxoid liposarcoma.

### Consent

Written informed consent was obtained from patient's parents for publication of this case report and any accompanying images.

## Competing interests

The author’s declared no potential competing interest with respect to the research, authorship, and/or publication of this article.

## Authors’ contributions

XJZ, KN and GJK performed the histological examination of the tumor and were major contributors to the writing of the manuscript. SZ, DFC and XHZ are the surgeons who operated on the patient and interpreted the patient data. All authors read and approved the final manuscript.
